# LncRNA FOXP4-AS1 is activated by PAX5 and promotes the growth of prostate cancer by sequestering miR-3184-5p to upregulate FOXP4

**DOI:** 10.1038/s41419-019-1699-6

**Published:** 2019-06-17

**Authors:** Xingcheng Wu, Yu Xiao, Yi Zhou, Zhien Zhou, Weigang Yan

**Affiliations:** 1 Department of Urology, Peking Union Medical College Hospital, Chinese Academy of Medical Sciences, Beijing, China; 2 Department of Pathology, Peking Union Medical College Hospital, Chinese Academy of Medical Sciences, Beijing, China

**Keywords:** Cancer, Cell biology

## Abstract

Prostate cancer (PCa) is one of the major men malignancies worldwide. Long noncoding RNAs (lncRNAs) have been reported as essential regulators in human cancers, including PCa. In the present study, lncRNA forkhead box P4 antisense RNA 1 (FOXP4-AS1) was found to be highly expressed in TCGA PCa samples. Upregulation of FOXP4-AS1 was further validated in 64 PCa tissues and predicted poor prognosis in patients with PCa. Functionally, high FOXP4-AS1 level was associated with increased cell proliferation and decreased cell apoptosis, indicating that FOXP4-AS1 exerted oncogenic functions in the tumorigenesis of PCa. Furthermore, FOXP4-AS1 was located in the cytoplasm of PCa cell lines and positively regulated FOXP4. LncRNAs can exert their functions by cooperating with their nearby genes. Mechanistically, FOXP4-AS1 post-transcriptionally regulated FOXP4 by acting as a competing endogenous RNA (ceRNA) in PCa to sponge miR-3184-5p. Considering the upregulation of both FOXP4-AS1 and its nearby gene FOXP4, we further detected the coactivator of FOXP4-AS1 and FOXP4. Mechanism analysis indicated that paired box 5 (PAX5) transcriptionally activated FOXP4-AS1 and FOXP4 in PCa. Collectively, we determined that PAX5-induced upregulation of FOXP4-AS1/FOXP4 axis promoted tumorigenesis of PCa.

## Introduction

Prostate cancer (PCa) is a commonly diagnosed malignant male cancer and is one of the prime reasons for cancer-related death^1^. Lacking of the biomarker in early diagnosis resulted in low overall survival of patients with PCa. Although numerous efforts have been made in exploring the novel therapeutic strategies, the prognosis of patients with advanced PCa remains unfavorable. Exploring the molecular mechanism associated with the tumorigenesis of PCa is of great significance for the early diagnosis or treatment of PCa.

Recent years, long noncoding RNAs (lncRNAs) that are mainly transcribed from the genomic intergenic regions have become a research focus in cancers transcriptome^2^. They have been demonstrated to be participants in various biological processes by different mechanisms^3,4^. The oncogenic or tumor-suppressive role of lncRNAs has been well characterized in tumorigenesis^5–7^. To date, some lncRNAs have been defined to be PCa specific, such as PCGEM1^8^, PRNCR1^9^, PCAT1^10^, and SChLAP1^11^. Nevertheless, investigating the function and mechanism of novel lncRNAs is necessary to find functional biomarkers in PCa progression. Searching from online database TCGA, we found top 30 upregulated lncRNAs in PCa samples. Among these candidates, lncRNA forkhead box P4 antisense RNA 1 (FOXP4-AS1) has been reported in osteosarcoma^12^ and colorectal cancer^13^. However, it is unclear whether FOXP4-AS1 exerted similar function in PCa tumorigenesis. Thus, we conducted in vitro and in vivo experiments to determine the function of FOXP4-AS1 in PCa growth.

To detect the potential mechanism pattern of lncRNA FOXP4-AS1 in PCa, we detected the cellular localization of FOXP4-AS1 in PCa cells and determined that FOXP4-AS1 was predominantly located in the cytoplasm of PCa cells, indicating that FOXP4-AS1 may regulate gene expression at post-transcriptional level. In term of post-transcriptional regulation, lncRNAs have been acknowledged to be competing endogenous RNAs (ceRNAs) by competitively binding to miRNA response elements to upregulate mRNAs^14–16^. On this basis, we carried out bioinformatics analysis and mechanism experiments to determine downstream genes of FOXP4-AS1. It was found that FOXP4-AS1 can regulate its nearby gene FOXP4 by sequestering miR-3184-5p.

Upregulation of genes in human cancers may be induced by transcription activation^17–19^. Here, we found several transcription factors in the common transcriptional region of FOXP4-AS1 and FOXP4. Further mechanism investigation was made to validate the effect of paired box 5 (PAX5) on the transcription activation of both FOXP4-AS1 and FOXP4. Collectively, the focus of this study is to explore the mechanism of PAX5-induced FOXP4-AS1/FOXP4 axis in PCa tumorigenesis.

## Materials and methods

### Bioinformatics analysis

Top 30 upregulated lncRNAs in PCa samples and the expression profile of FOXP4-AS1 in PCa samples were acquired from TCGA dataset (http://gepia.cancer-pku.cn/index.html). UCSC (http://genome.ucsc.edu/) was applied to search the nearby gene of FOXP4-AS1. DIANA tools (http://carolina.imis.athena-innovation.gr/diana_tools/web/index.php?r=site%2Ftools) and the starbase database (http://starbase.sysu.edu.cn/) were used to predict the miRNAs that had complementary base paring with both FOXP4-AS1 and FOXP4. The DNA motif of PAX5 and five putative binding sites of PAX5 in FOXP4-AS1 or FOXP4 promoter were obtained from JASPAR (http://jaspar.genereg.net/).

### Patient specimens and cell culture

Sixty-four matched PCa and adjacent nontumor prostate tissues were collected from patients with PCa who received treatment at The Fourth Hospital of Harbin Medical University from May 2012 to June 2017. After resection, all tissues were snap-frozen in liquid nitrogen and stored at −80 °C. The written informed consents were provided by all the participants. Our study was approved by the Research Ethics Committee of the The Fourth Hospital of Harbin Medical University and complied with the Declaration of Helsinki. According to the median of the expression level of FOXP4-AS1, miR-3184-5p or FOXP4, 64 PCa tissues were classified into high-expression group (*n* = 32) and low-expression group (*n* = 32).

Human PCa cell line (PC-3, DU145, VCaP, and LNCaP) and human normal prostate epithelial cell line (RWPE-1) were bought from the American Type Culture Collection (Rockville, MD, USA). Cell culture was conducted in Dulbecco’s Modified Eagle Medium containing 10% fetal bovine serum (HyClone, Logan, UT, USA), 1% penicillin-streptomycin (HyClone) at 37 °C with 5% CO_2_.

### Quantitative real-time PCR

Total RNA was extracted from tissue samples using TRIzol reagent (Thermo Fisher Scientific, Waltham, MA, USA) following the user guide. RNA was converted into cDNA using random primers and Reverse Transcription Kit (Toyobo, Osaka, Japan). Nanodrop 1000 spectrophotometer (Thermo Fisher Scientific, Waltham, MA, USA) and 1.5% denaturing agarose gels were used to measure RNA concentration and purity. The primers are shown in Supplementary Table 1. To evaluate the expression of lncRNA or mRNA, qRT-PCR was conducted using SYBR Green PCR Master Mix (Takara, Kyoto, Japan) with Roche Real-Time PCR system (Roche, USA). Relative expression was normalized to GAPDH or U6 and was determined by 2^−ΔΔCt^ method.

### Cell transfection

For cell transfection, LNCaP and PC-3 cell lines at 70–80% confluence were planted in six-well plates. For overexpression of genes, pcDNA3.1 vectors (Genechem Company, Shanghai, China) were subcloned with FOXP4-AS1, FOXP4, or PAX5 (pcDNA-FOXP4-AS1, pcDNA-FOXP4, or pcDNA-PAX5). Empty pcDNA3.1 vector was used as negative control. The short hairpin RNAs (shRNAs) against FOXP4-AS1, FOXP4, and PAX5 (sh-FOXP4-AS1#1/2/3, sh-FOXP4#1/2/3, and sh-PAX5#1/2/3) were designed and synthesized by GenePharma (Shanghai, China). To overexpress miR-3184-5p, miR-3184-5p mimics and miR-NC (GenePharma) were transfected into PCa cell lines. Whereas miR-3184-5p inhibitor and its negative control anti-miR-NC (GenePharma) were transfected into PCa cells for silencing of miR-3184-5p. Similarly, STAT5A, EBF1, RAD21, and RUNX3 were overexpressed by subcloning the whole sequence of them into pcDNA3.1 vectors (Genechem). shRNAs targeting STAT5A, EBF1, RAD21, and RUNX3 were synthesized by Genechem were used for knockdown of them. Lipofectamine 2000 (Invitrogen, Carlsbad, CA, USA) was used for transfection following the standard method. After 48 h, cells were reaped for subsequent experiments.

### Cell proliferation assays

A total of 5 × 10^3^ transfected LNCaP and PC-3 cell lines were harvested and put into 96-well plates. Cell viability was analyzed using 10 μL of Cell Counting Kit-8 reagent (CCK-8, DOJINDO, Kumamoto, Japan). The absorbance at 450 nm was evaluated by a microplate reader when the reagent reacted for 24, 48, 72 and 96 h at room temperature. All experimental procedures were conducted in triplicate.

For colony formation assay, LNCaP and PC-3 cell lines were plated in six-well plates at 500 cells per well for 2 weeks. After fixation in 4% paraformaldehyde for 10 min, cells were subjected to 0.1% crystal violet for 30 min. Finally, colonies were counted and recorded. Experiments were repeated at least three times.

For EdU incorporation assay, LNCaP and PC-3 cell lines on sterile coverslips were seeded in 24-well plates. Cell proliferation was detected by the EdU incorporation assay kit (Ribobio, Guangzhou, China). The stained cells were visualized by laser confocal microscopy (FV300, Olympus, Tokyo, Japan). Nucleus was double-stained with EdU and 4′,6-diamidino-2-phenylindole (DAPI, Beyotime, Shanghai, China) as positively proliferative cells. All experiments were repeated in triplicate.

### JC-1 fluorescence analysis

Cell apoptosis was assessed by examine the mitochondrial transmembrane potential (∆Ψ). LNCaP and PC-3 cell lines were seeded in 96-well plates (1 × 10^5^ cells/well) and incubated overnight. Afterward, cells were subjected to Cis and/or mdivi-1, with or without 2 mM NAC or 0.5 mM TEMPOL in serum-free medium for 18 h. The cationic, lipophilic dye 5,5′,6,′-tetrachloro-1,1′,3,3′ tetraethylbenzimidazolyl carbocyanine iodide (JC-1) was commercially obtained from Cayman Chemical (Ann Arbor, MI, USA). After centrifugation, cells were loaded in JC-1 dye for half an hour, following washing and incubation in assay buffer. ∆Ψ was detected by a fluorescent plate reader. A fluorescence microscope was used to capture Gemini XPS and images. Each experimental procedure was repeated at least three times

### Caspase-3 activity analysis

To detect caspase-3 activity, an EnzCheck^®^ Caspase-3 Assay kit#1 was purchased from Molecular Probes (Eugene, OR, USA). The rinsed cells were lysed in cell lysis buffer on ice for 10 min, followed by centrifugation. The supernatant was transferred into microplate wells and subjected to Z-DEVD-AMC as caspase-3 substrate. Gemini XPS fluorescent plate reader was applied to measure fluorescent signals at a wavelength of 405 nm. Each apoptotic assay was performed at least three times.

### In vivo experiment and immunohistochemical staining

Male BALB/C athymic nude mice (aged 6 weeks) were bought from the National Laboratory Animal Center (Beijing, China) and preserved in an SPF-grade pathogen-free animal laboratory. Animal experiment was conducted strictly in light with the protocol approved by the Animal Research Ethics Committee of The Fourth Hospital of Harbin Medical University and the principles of the Declaration of Helsinki. A total of 1 × 10^7^ transfected LNCaP and PC-3 cell lines were injected subcutaneously around the left flank of nude mice (three mice per group). Tumor volumes were calculated with the following equation: 0.5 × length × width^2^ every 4 days. Four weeks later, mice were killed. Tumors were excised, weighed, and snap-frozen at −80 °C for subsequent analysis.

### Ki-67 staining

The excised tumor tissue specimens were put in formalin, fixed and paraffin-embedded. After deparaffinating and rehydrating, tissues were incubated with antibody against Ki-67 (Santa Cruz Biotechnology, Dallas, TX, USA) at 4 °C overnight. Tissues were stained with diaminobenzidine and observed under light microscopy.

### Western blotting

Protein samples from tissues or cells were acquired using RIPA lysis buffer (Beyotime, Beijing, China) and separated on 10% SDS-PAGE. After transferring onto PVDF membranes, proteins were blocked with 5% solution of nonfat milk for 2 h. Subsequently, samples were treated with primary antibodies against FOXP4 (ab251688, Abcam, Cambridge, UK) and GAPDH (ab8245, Abcam) along with the corresponding secondary antibodies conjugated to horseradish peroxidase. The signals of membranes were measured by ECL Substrate (Pierce, Rockford, IL). GAPDH was used as an internal reference.

### Subcellular fractionation assay

The subcellular fractionation assay was carried out by the use of PARIS^™^ Kit (Invitrogen, USA) following the supplier’s suggestion. LNCaP and PC-3 cell lines were washed in prechilled PBS for three times and lysed in cell fractionation buffer. The supernatant was collected. The cell lysates were placed in cell fractionation buffer and centrifuged. Cell disruption buffer was added to lyse cell nuclei. The isolated RNA was assessed by quantitative real-time PCR. GAPDH and U6 were used as cytoplasmic or nuclear fractionation indicators.

### Fluorescence in situ hybridization (FISH)

After fixation with 4% formaldehyde and washing with PBS, PC-3 cells were treat with Pepsin (1% in 10 mmol/l HCl) and was dehydrated by ethanol. Then, dried cells were mixed in a hybridization buffer by using 40 nmol/l of the FISH probe (FOXP4-AS1 lncRNA) and incubated at 80 °C for 120 s. After being stayed at 55 °C for 120 min, the slides were washed and dehydrated again. Finally, the slides were detected by Prolong Gold Antifade Reagent with DAPI. Fluorescence-conjugated FOXP4-AS1 probes were designed and synthesized by Ribobio Company (Guangzhou, China).

### RNA immunoprecipitation assay (RIP)

For Ago 2-RIP assay, the Magna RIP RNA-Binding Protein Immunoprecipitation Kit was purchased from Millipore (Billerica, MA, USA) in line with the user guide. LNCaP and PC-3 cell lines were washed in precold PBS and lysed in RIP buffer at 4 °C for half an hour. Cell lysates were treated with magnetic beads conjugated to antibodies against Ago 2 (Millipore) or normal mouse immunoglobulin G (IgG; Millipore). Immunoprecipitated RNA was extracted for quantitative real-time PCR. For MS2-RIP assay, cells treated with pMS2-GFP were cotransfected with pcDNA3.1-MS2 and pcDNA3.1-FOXP4-AS1-MS2 for 48 h. Thereafter, cells were incubated with GFP antibody (Roche Diagnostics GmbH, Mannheim, Germany) and the Magna RIP RNA-Binding Protein Immunoprecipitation Kit in accordance with the guidebook for user. After collecting and lysing, cell suspension was cultured with magnetic beads. The antibody was added and incubated overnight. After RNA purification, the isolated RNA was detected by quantitative real-time PCR to quantify the presence of the binding targets.

### Luciferase reporter assay

The amplified FOXP4-AS1 or 3′-UTR of FOXP4 was subcloned into the firefly plasmids in pmirGLO luciferase vector (GeneChem, Shanghai, China). The wild type of FOXP4-AS1 or FOXP4 3′-UTR (FOXP4-AS1-WT or FOXP4-WT) was constructed. The site-directed mutation of miR-3184-5p binding sites in FOXP4-AS1 or FOXP4 3′-UTR (FOXP4-AS1-MUT or FOXP4-MUT) was generated using the GeneTailor™ Site-Directed Mutagenesis System (Invitrogen, Carlsbad, CA, USA). LNCaP and PC-3 cell lines were separately cotransfected with aforementioned plasmids and miR-3184-5p mimics or miR-NC. At last, a Dual-Luciferase Reporter Assay System (Promega) was used to evaluate luciferase activity. For FOXP4-AS1 or FOXP4 promoter luciferase assay, cells were placed into 24-well plates and cotransfected with the plasmids containing the putative binding sites of PAX5 to FOXP4-AS1 or FOXP4 promoter. All these plasmids were simultaneously subcloned into pGL3 vector (Promega Corporation, Fitchburg, WI, USA) and established. Each procedure of these experiments was repeated independently for three times.

### Chromatin immunoprecipitation assay (ChIP)

The ChIP assay was performed by the use of SimpleChIP^®^ Enzymatic Chromatin IP Kit (CST, Danvers, MA, USA) following the supplier’s protocol. The crosslinked chromatin DNA was broken to 200 to 1000 bp through enzymatic digestion. Then, the chromatin was immunoprecipitated with antibodies against PAX5 and IgG. Precipitated chromatin DNA was recovered by magnetic beads and analyzed by quantitative real-time PCR. All samples were prepared in triplicate.

### Statistical analysis

All experimental data were presented as the mean ± standard deviation of three or more repetitive experiments. SPSS 13.0 (SPSS, Inc., Chicago, IL, USA) and GraphPad PRISM 6 (GraphPad, San Diego, CA, USA) were used for statistical analysis. Kaplan–Meier analysis was utilized to estimate the overall survival rate of patients with PCa. Correlation was analyzed with Pearson’s correlation method. Differences between groups were analyzed by Student's *t*-test or one-way ANOVA. Differences were considered to be significant when *P* < 0.05.

## Results

### Upregulation of FOXP4-AS1 in PCa samples is correlated with unfavorable patients’ prognosis

**Fig. 1:**
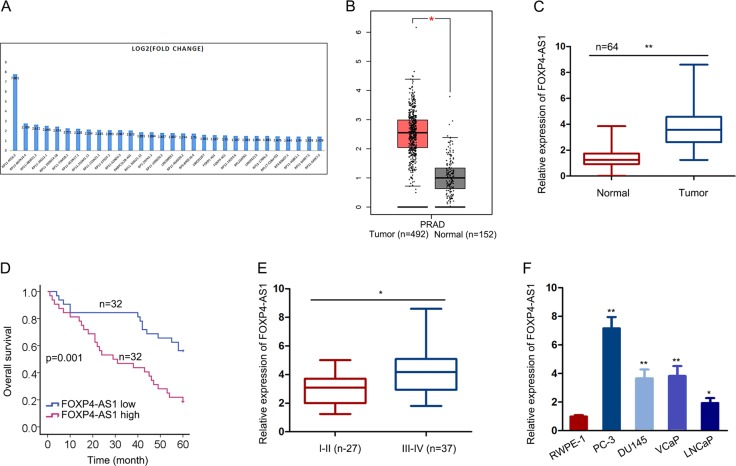
Upregulation of FOXP4-AS1 in PCa samples is correlated with unfavorable patients’ prognosis. **a** Thirty lncRNAs that are upregulated in TCGA PCa samples were listed. **b** FOXP4-AS1 expression prolife in PCa and normal tissues was obtained from the TCGA database. **c** The expression level of FOXP4-AS1 was examined in PCa samples and adjacent samples collected from 64 patients with PCa. **d** Overall survival of 64 patients with PCa with high or low FOXP4-AS1 level was analyzed. **e** The expression level of FOXP4-AS1 in patients with PCa with early or advanced tumor stage. **f** Expression level of FOXP4-AS1 in PCa cell lines and one human normal prostate epithelial cell RWPE-1. *P < 0.05, **P < 0.01

Based on the TCGA database, top 30 upregulated lncRNAs in PCa samples were searched out and illustrated in Fig. 1a. Among which, FOXP4-AS1 has been reported as an oncogene in colorectal cancer and osteosarcoma. However, it is unknown that whether FOXP4-AS1 exerted function in PCa. The expression profile of FOXP4-AS1 in TCGA PCa samples were detected and shown (Fig. 1b). Moreover, the expression level of FOXP4-AS1 was examined in PCa samples and adjacent samples collected from 64 patients with PCa. As expected, FOXP4-AS1 was expressed at a higher level in PCa samples compared to adjacent normal samples (Fig. 1c). To analyze the prognostic potential of FOXP4-AS1 in patients with PCa, Kaplan–Meier surviving curves were generated. After analysis, we determined that high level of FOXP4-AS1 was closely associated with the low overall survival rate of patients with PCa (Fig. 1d). Furthermore, the relative higher level of FOXP4-AS1 was observed in patients in advanced stage rather than those in early stage (Fig. 1e). Consistently, the expression level of FOXP4-AS1 was found to be higher in PCa cell lines compared to human normal prostate epithelial cell RWPE-1 (Fig. 1f). These data indicated that FOXP4-AS1 might be a participant in tumorigenesis of PCa.

### FOXP4-AS1 facilitated PCa cell growth in vitro

**Fig. 2:**
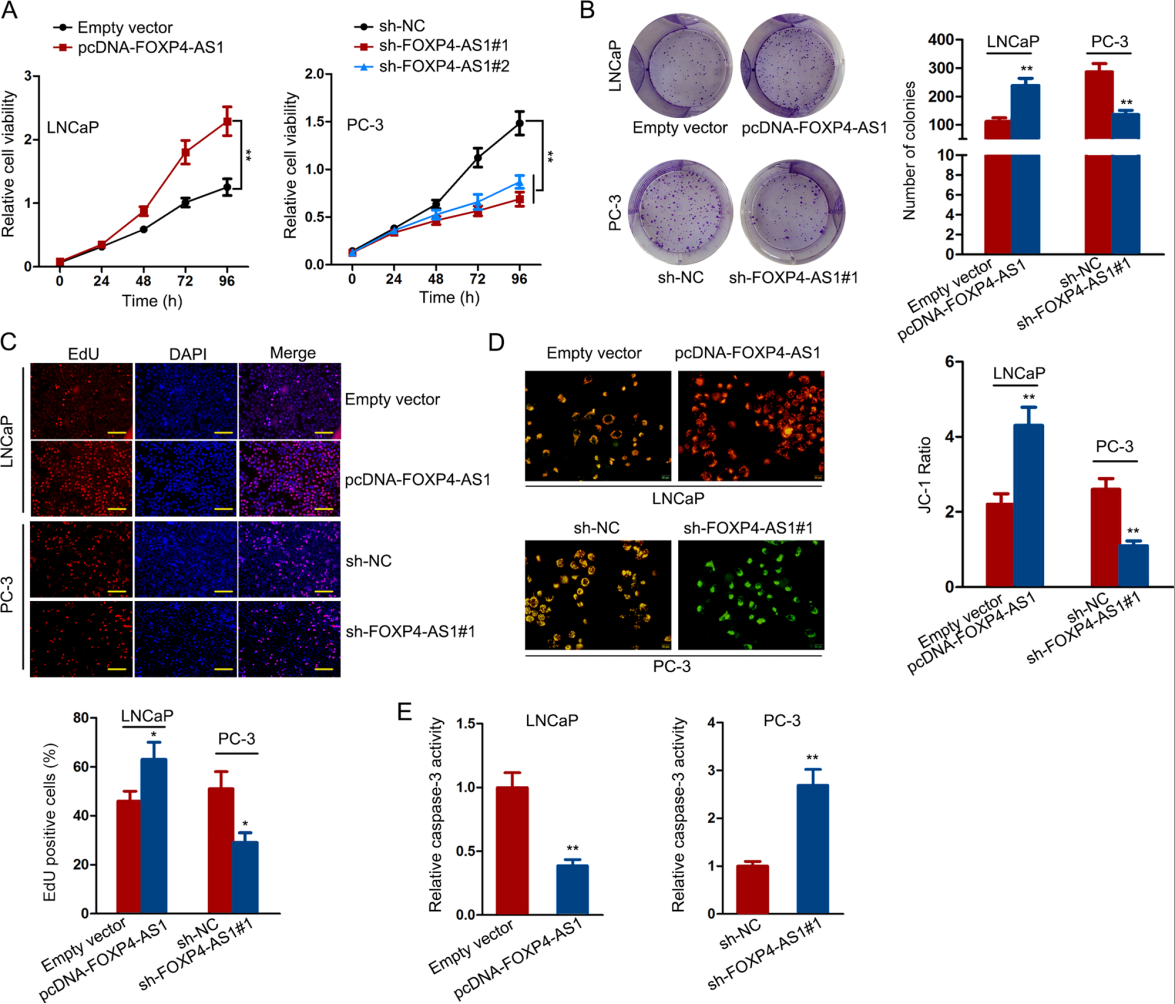
FOXP4-AS1 facilitated PCa cell growth in vitro. **a** The CCK-8 assay was used to detect cell viability after overexpression or knockdown of FOXP4-AS1. **b**, **c** Proliferative ability of LNCaP cell transfected with FOXP4-AS1 expression vector or PC-3 cell transfected with sh-FOXP4-AS1 was assessed by the colony formation assay and EdU assay. Scale bar for EdU staining: 100 μm. **d** Membrane potential in FOXP4-AS1-overexpressed or -downregulated PCa cells was measured by JC-1 staining. Scale bar = 200 μm. **e** Caspase-3 activity was tested in two PCa cells that were separately transfected with FOXP4-AS1 expression vector or sh-FOXP4-AS1#1. *P < 0.05, **P < 0.01

To explore the role of FOXP4-AS1 in PCa cellular function, we designed functional assays in two different PCa cell lines. According to the data in Fig. 1g, LNCaP cell exhibited the relative lowest expression level of FOXP4-AS1, while PC-3 cell presented the highest level of FOXP4-AS1 expression. Thus, we overexpressed FOXP4-AS1 in LNCaP cell but silenced it in PC-3 cell (Supplementary Fig. 1A). At first, we performed the CCK-8 assay to detect the viability of cells with high or low level of FOXP4-AS1. The results indicated that overexpression of FOXP4-AS1 efficiently enhanced cell viability, whereas downregulation of FOXP4-AS1 led to the decreased cell viability (Fig. 2a). Cell viability was suppressed more efficient when cells were transfected with sh-FOXP4-AS1#1. Therefore, sh-FOXP4-AS1#1 was chosen for subsequent experiments. Through colony formation and EdU assays, we determined that overexpression of FOXP4-AS1 markedly promoted cell proliferation, while knockdown of FOXP4-AS1 led to an opposite result (Fig. 2b, c). Then, we explored whether FOXP4-AS1 regulated cell proliferation by inducing cell apoptosis. According to JC-1 staining, we supposed that high expression level of FOXP4-AS1 correlated with increased membrane potential, indicating the inhibitory effect of FOXP4-AS1 on the early apoptosis of PCa cells (Fig. 2d). In addition, we tested caspase-3 activity in two PCa cells that were separately transfected with FOXP4-AS1 expression vector or sh-FOXP4-AS1#1. It was found that FOXP4-AS1 negatively regulated caspase-3 activity in PCa cells (Fig. 2e). Therefore, we confirmed that ectopic expression of FOXP4-AS1 affected PCa proliferation and apoptosis.

### FOXP4-AS1 promoted PCa tumor growth in vivo

**Fig. 3:**
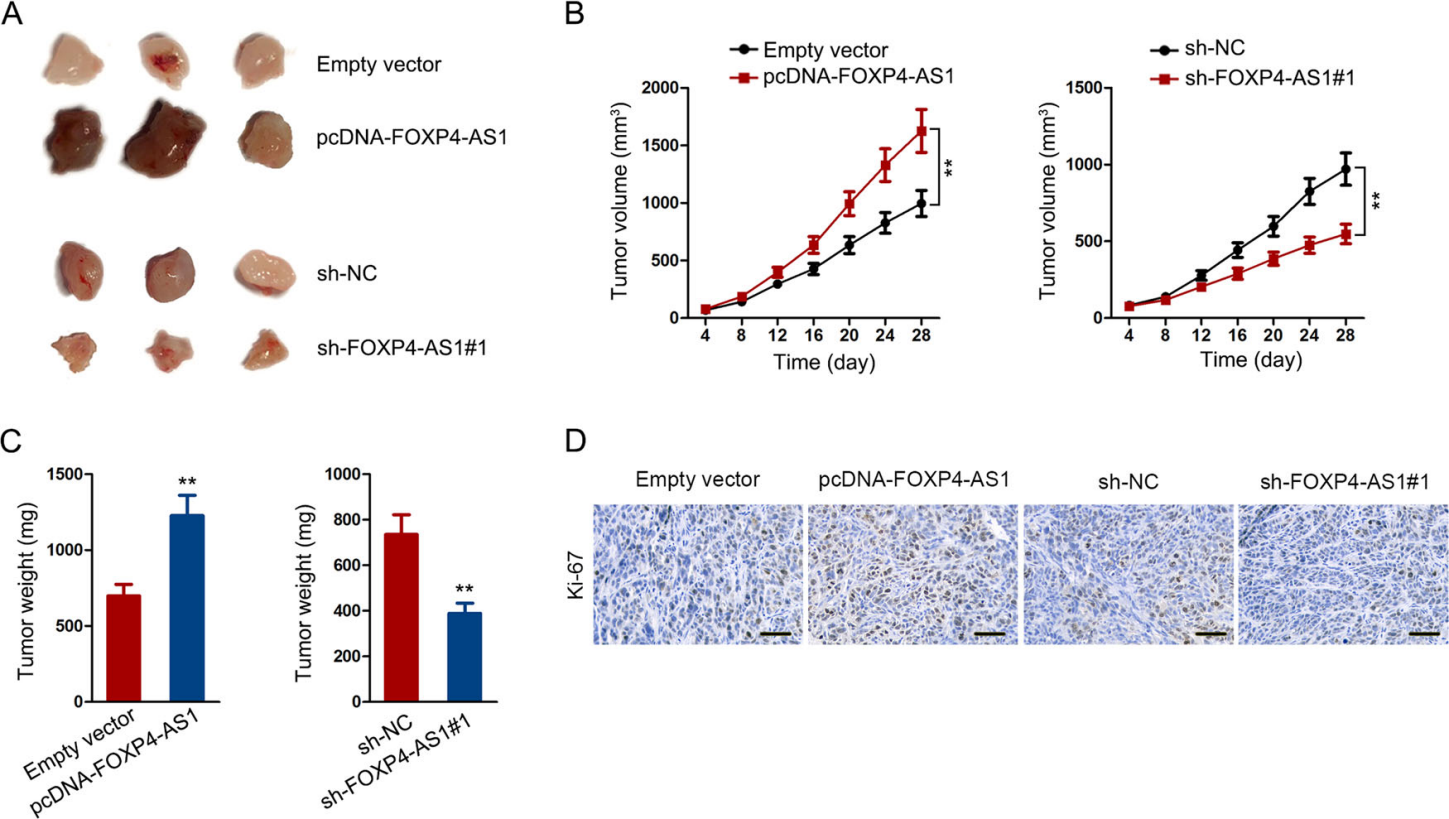
FOXP4-AS1 promoted PCa tumor growth in vivo. **a** Tumors derived from FOXP4-AS1-overexpressed LNCaP cell or FOXP4-AS1-downregulated PC-3 cell was resected and shown. **b**, **c** Tumor volume and tumor weight in different groups were quantified and illustrated. **d** Immuohistochemical staining of ki-67 positivity in tumors of different groups. Scale bar = 50 μm. **P < 0.01

Furtherly, animal study was carried out to demonstrate the role of FOXP4-AS1 in regulating PCa growth. LNCaP cell stably transfected with pcDNA-FOXP4-AS1 or empty vector and PC-3 cell stably transfected with sh-FOXP4-AS1#1 or sh-NC were separately injected into nude mice. After 28 days, tumors were removed and calculated. As presented in Fig. 3a, tumors derived from cell transfected with pcDNA-FOXP4-AS1 were bigger than those derived from cell transfected with empty vector. In addition, tumors in sh-FOXP4-AS1#1 group were smaller than those in sh-NC group (Fig. 3a). Consistently. Same tendencies were observed in tumor volume and tumor weight (Fig. 3b, c). Finally, immuohistochemical staining indicated that ki-67 positivity in FOXP4-AS1 overexpression group was significantly higher in NC group. However, sh-FOXP4-AS1#1 group exhibited low ki-67 positivity compared to sh-NC group (Fig. 3d).

### FOXP4 was positively regulated by FOXP4-AS1 and exerted oncogenic property in PCa

**Fig. 4:**
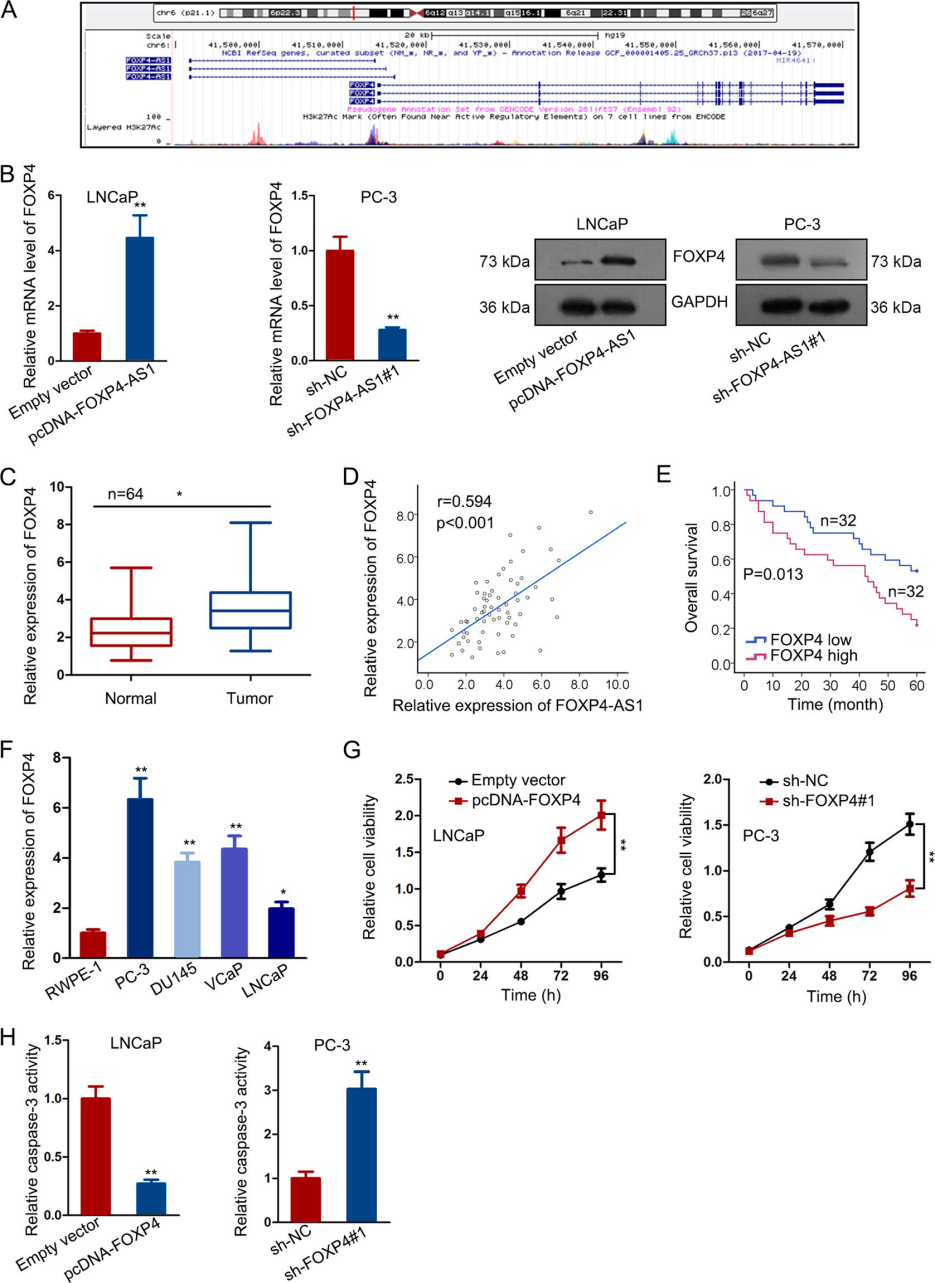
FOXP4 was positively regulated by FOXP4-AS1 and exerted oncogenic property in PCa. **a** UCSC data revealed that FOXP4 is a nearby gene of FOXP4-AS1. **b** The mRNA and protein level of FOXP4 in cells transfected with pcDNA-FOXP4-AS1 or sh-FOXP4-AS1 were measured by qRT-PCR and western blotting. **c** Upregulation of FOXP4 in PCa samples. **d** Positive expression association between FOXP4-AS1 and FOXP4 in PCa samples was analyzed by Pearson correlation analysis. **e** Kaplan–Meier analysis of patients with PCa with high or low expression level of FOXP4. **f** Relative high level of FOXP4 was detected in PCa cells and one normal control cell. **g** Cell viability was examined in response to the overexpression or knockdown of FOXP4 by the CCK-8 assay. **h** Caspase-3 activity was increased in LNCaP cell transfected with FOXP4 expression vector but was increased in PC-3 cell transfected with sh-FOXP4#1. **P < 0.01

Using online bioinformatics analysis tool UCSC, we found that FOXP4 is a nearby gene of FOXP4-AS1 (Fig. 4a). FOXP4 has been reported in human cancers due to its oncogenic property. Through qRT-PCR and western blotting analyses, we found that FOXP4-AS1 could positively regulate both mRNA and protein level of FOXP4 in PCa cells (Fig. 4b). Furthermore, we found that FOXP4 was upregulated in PCa samples (Fig. 4c), which was consistent with FOXP4-AS1 (Fig. 4d). Similarly, we made survival analysis to determine whether FOXP4 was correlated with patients’ survival. It was found that patients in FOXP4 high-expression group had lower overall survival rate than those in FOXP4 low-expression group (Fig. 4e). The relative high level of FOXP4 was detected in PCa cells compared with the normal control cell (Fig. 4f). To investigate the potential of FOXP4 in regulating PCa cell growth, we overexpressed or silenced it in indicated PCa cells by transfecting with FOXP4 expression vector or specific shRNA (Supplementary Fig. 1B). Then, CCK-8 and caspase-3 activity test were used to evaluate the importance of FOXP4 in PCa cell proliferation and apoptosis. Interestingly, overexpression of FOXP4 promoted cell proliferation, while knockdown of FOXP4 had inhibitory effect on cell proliferation (Fig. 4g). In addition, the caspase-3 activity was decreased in LNCaP cell transfected with FOXP4 expression vector but was increased in PC-3 cell transfected with sh-FOXP4#1 (Fig. 4h). These data showed that FOXP4 might be cooperated with FOXP4-AS1 to exert function in PCa.

### FOXP4-AS1 acted as the molecular sponge of miR-3184-5p

**Fig. 5:**
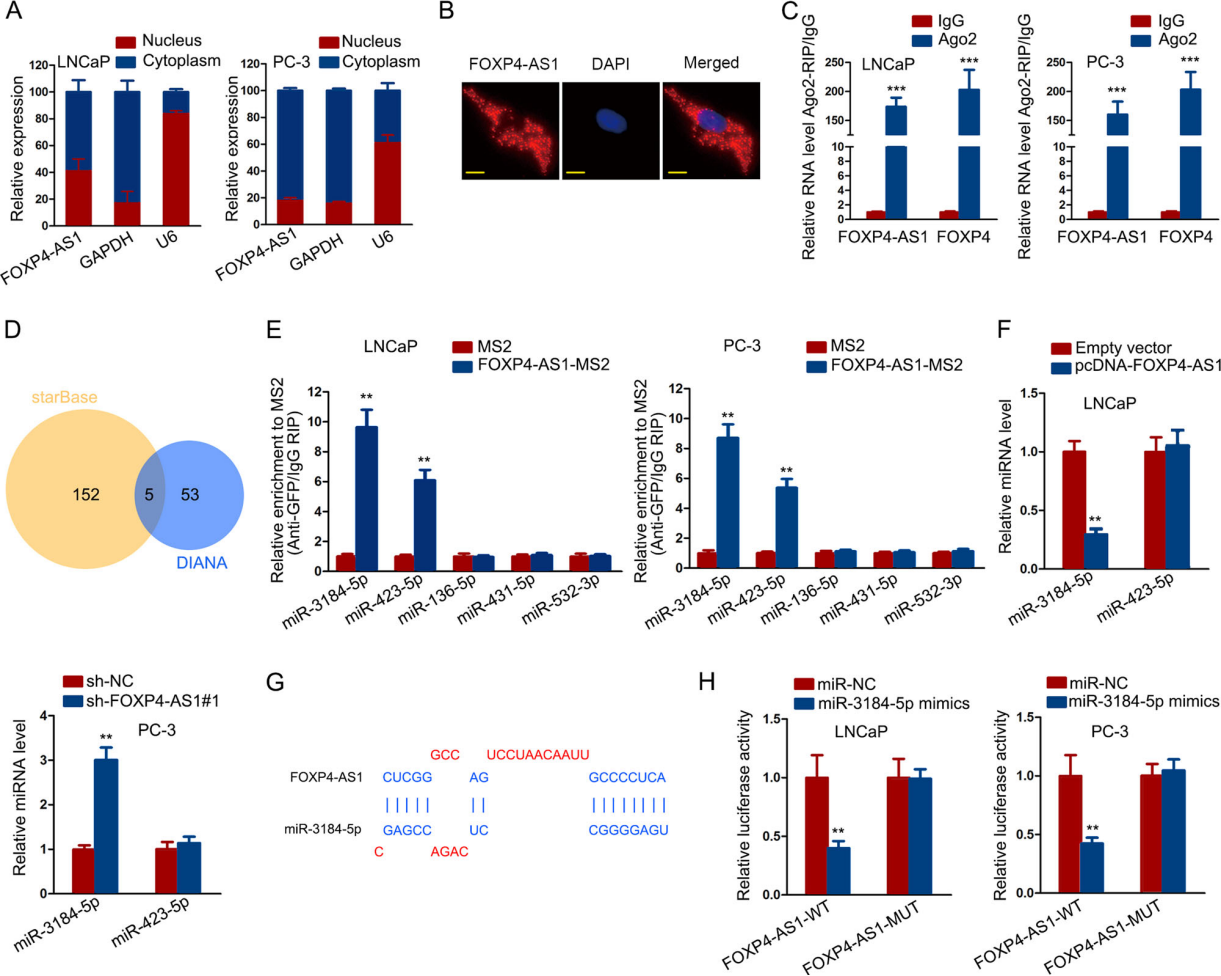
FOXP4-AS1 acted as the molecular sponge of miR-3184-5p. **a**, **b** FOXP4-AS1 was predominantly located in the cytoplasm of PCa cells. Scale bar = 200 μm. **c** Ago 2-RIP revealed that both FOXP4-AS1 and FOXP4 were enriched in Ago 2 containing beads. **d** Five miRNAs that had complementary base paring with both FOXP4-AS1 and FOXP4 were predicted from starBase and DIANA. **e** Enrichment of five miRNAs in MS2 with or without FOXP4-AS1. **f** miR-3184-5p and miR-423-5p were detected in cells transfected with FOXP4-AS1 expression vector or sh-FOXP4-AS1#1. **g** The predicted binding sequence between FOXP4-AS1 and miR-3184-5p. **h** The luciferase reporter assay was conducted in cells transfected with miR-3184-5p mimics or miR-NC to examine the luciferase activity of FOXP4-AS1-WT and FOXP4-AS1-MUT. *P < 0.05, **P < 0.01

LncRNAs can exert functions by transcriptionally or post-transcriptionally activating their downstream genes. To investigate the regulatory pattern of FOXP4-AS1 in PCa, we detected its cellular fractionation in two PCa cells through the subcellular fractionation assay and FISH assay. Both experimental results indicated that FOXP4-AS1 was predominantly located in the cytoplasm of PCa cells (Fig. 5a, b). Therefore, we identified the post-transcriptional regulation of FOXP4-AS1 in PCa. CeRNA network is a common post-transcriptional regulatory pattern of lncRNAs. Combining with above data, we hypothesized that FOXP4-AS1 might upregulate FOXP4 by sequestering a miRNA. Next, we performed the Ago 2-RIP assay in two PCa cells. The results showed that both FOXP4-AS1 and FOXP4 were enriched in Ago 2 containing beads, suggesting the probability of the involvement of FOXP4-AS1 and FOXP4 in ceRNA network (Fig. 5c). Based on bioinformatics analysis, we searched out five miRNAs that had complementary base paring with both FOXP4-AS1 and FOXP4 (Fig. 5d). Subsequently, the MS2-RIP assay was carried out to demonstrate the binding of these five potential miRNAs to FOXP4-AS1. As a result, miR-3184-5p and miR-423-5p showed the strongest affinity to FOXP4-AS1-MS2 beads (Fig. 5e). Then, we examined the expression level of these two miRNAs in cells transfected with FOXP4-AS1 expression vector or shRNAs. The results showed that miR-3184-5p was efficiently downregulated by overexpressing FOXP4-AS1 but was upregulated by silencing FOXP4-AS1 (Fig. 5f). The predicted binding sequence between FOXP4-AS1 and miR-3184-5p was shown (Fig. 5g). To change the expression level of miR-3184-5p in PCa cells, miR-3184-5p inhibitor and miR-3184-5p mimics were separately transfected into LNCaP and PC-3 cells. Luciferase activity analysis revealed the effect of miR-3184-5p mimics on the luciferase activity of reporter containing wild type FOXP4-AS1 (FOXP4-AS1-WT) or mutant type FOXP4-AS1 (FOXP4-AS1-MUT). The results suggested that miR-3184-5p mimics efficiently decreased the luciferase activity of FOXP4-AS1-WT vector (Fig. 5h). All these experimental results indicated that FOXP4-AS1 might act as a ceRNA to regulate miR-3184-5p and FOXP4.

### FOXP4 is the target of miR-3184-5p in PCa

**Fig. 6:**
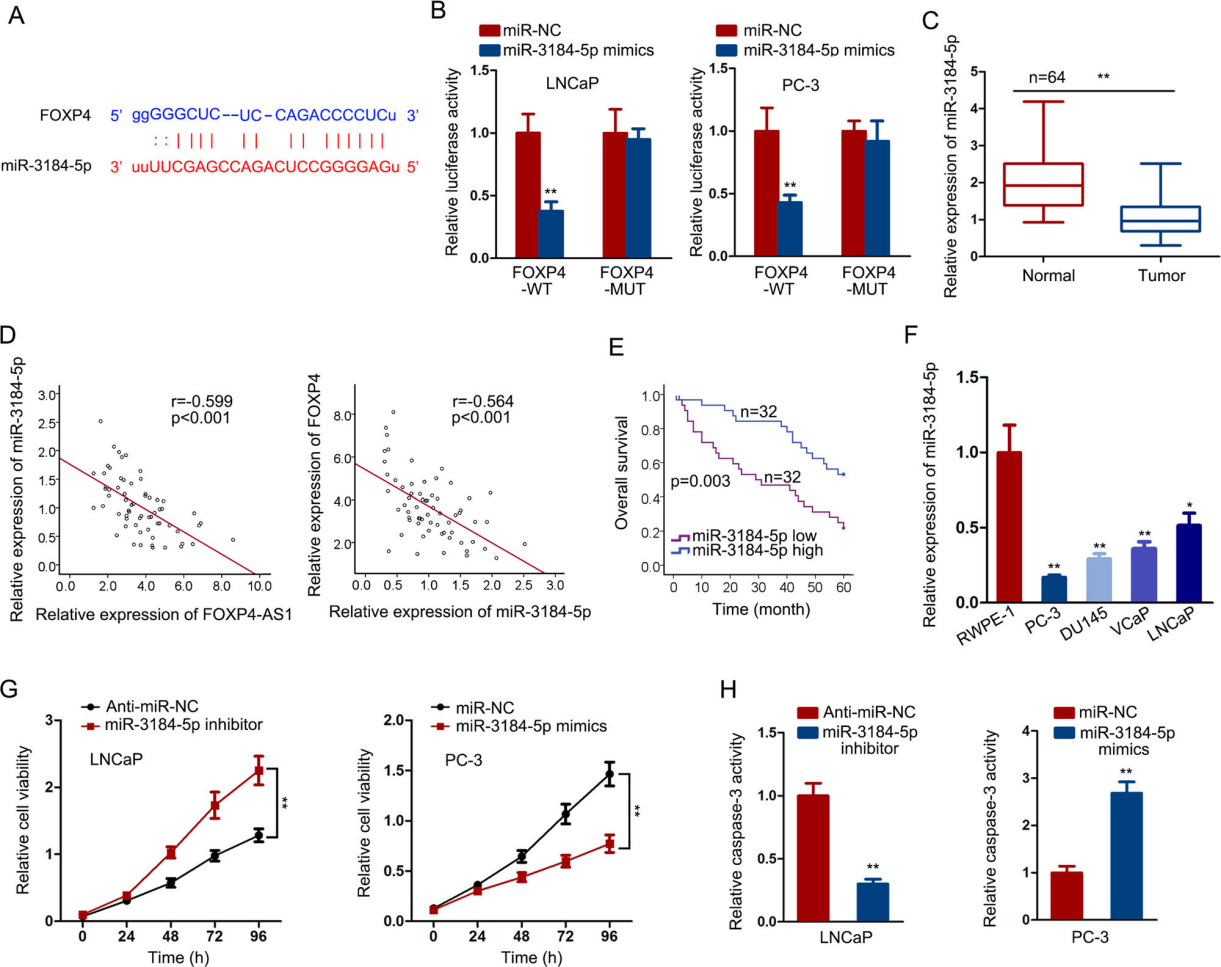
FOXP4 is the target of miR-3184-5p in PCa. **a** The binding sequence of miR-3184-5p to FOXP4 3′UTR. **b** Luciferase activity of reporters containing wild type FOXP4 (FOXP4-WT) or mutant type FOXP4 (FOXP4-MUT) in cells transfected with miR-3184-5p mimics. **c** MiR-3184-5p expression in PCa samples and normal samples. **d** Expression association between miR-3184-5p and FOXP4-AS1 or FOXP4 in PCa samples. **e** Kaplan–Meier analysis of patients with PCa with high or low expression level of miR-3184-5p. **f** MiR-3184-5p expression in PCa cell lines and normal cell line. **g**, **h**. The effect of miR-3184-5p inhibitor or mimics on cell proliferation or apoptosis. **P < 0.01, ***P < 0.001

Likewise, we performed experiments to demonstrate the interaction between miR-3184-5p and FOXP4. At first, the binding sequence of miR-3184-5p to FOXP4 3′UTR was predicted and obtained (Fig. 6a). Then, we examined the luciferase activity of reporter containing wild-type FOXP4 (FOXP4-WT) or mutant-type FOXP4 (FOXP4-MUT) in cells transfected with miR-3184-5p mimics. The results suggested that the luciferase activity of FOXP4-WT vector but not FOXP4-MUT vector was decreased by miR-3184-5p mimics (Fig. 6b). Subsequently, we tested the expression level of miR-3184-5p in PCa samples. Unsurprisingly, miR-3184-5p exhibited relative low level in PCa samples, which was negative with FOXP4-AS1 or FOXP4 (Fig. 6c, d). Kaplan–Meier analysis further revealed the positive correlation between miR-3184-5p expression and the overall survival rate of patients with PCa (Fig. 6e). Compared with normal cell line, PCa cell lines exhibited relative low expression level of miR-3184-5p (Fig. 6f). Functionally, we found that inhibition of miR-3184-5p had a positive effect on cell proliferation but had a negative effect on cell apoptosis. On the other hand, upregulation of miR-3184-5p led to growth inhibition by suppressing cell proliferation and promoting cell apoptosis (Fig. 6g, h). Taken together, FOXP4 exerted oncogenic function in PCa and participated in a ceRNA pathway by cooperating with FOXP4-AS1 and FOXP4.

### FOXP4 involved in FOXP4-AS1-mediated PCa cell growth

**Fig. 7:**
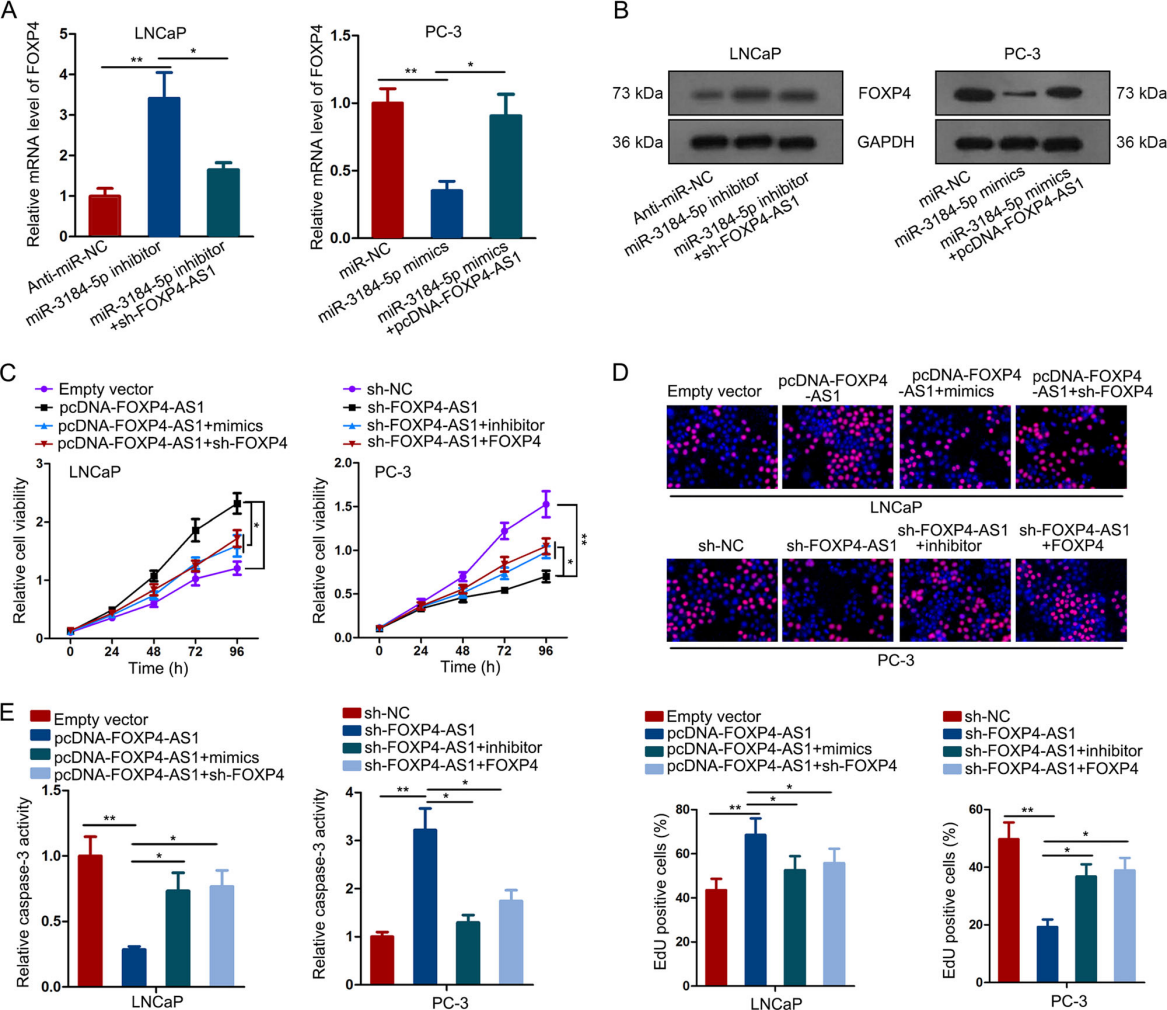
FOXP4 involved in FOXP4-AS1-mediated PCa cell growth. **a**, **b** The effect of miR-3184-5p inhibitor or mimics on FOXP4 expression was attenuated by FOXP4-AS1. **c**, **d** Cell proliferation was examined after transfected with indicated plasmids. Scale bar = 100 μm. **e** The effect of miR-3184-5p or FOXP4 on FOXP4-AS1-mediated cell apoptosis. *P < 0.05, **P < 0.01

Then, we analyzed the expression level of FOXP4 in indicted PCa cells. It was found that mRNA and protein level of FOXP4 were negatively regulated by miR-3184-5p. And the effect of miR-3184-5p inhibitor or mimics on FOXP4 expression was attenuated by FOXP4-AS1 (Fig. 7a, b). These data further demonstrated the regulatory network of FOXP4-AS1/miR-3184-5p/FOXP4. According to above data, we confirmed that FOXP4-AS1 can exert function in PCa by sequestering miR-3184-5p to upregulate its nearby gene FOXP4. To strengthen our viewpoint, we conducted rescue assays in two PCa cells. Cell proliferation assays demonstrated that miR-3184-5p mimics or sh-FOXP4 reversed the effect of FOXP4-AS1 overexpression on LNCaP cell proliferation. Moreover, inhibition of miR-3184-5p expression or overexpression of FOXP4 attenuated the impact of sh-FOXP4-AS1 on PC-3 cell proliferation (Fig. 7c, d). Caspase-3 activity was also tested in cells transfected with indicated plasmids. The experimental results proved that upregulation of miR-3184-5p or knockdown of FOXP4 reversed the inhibitory effect of pcDNA-FOXP4-AS1 on cell apoptosis. However, the positive effect of silenced FOXP4-AS1 on cell apoptosis was attenuated in PC-3 cell cotransfected with miR-3184-5p inhibitor or FOXP4 expression vector (Fig. 7e). Collectively, we confirmed that FOXP4-AS1 promoted PCa growth by regulating miR-3184-5p/FOXP4 axis.

### PAX5 transcriptionally activated FOXP4-AS1 and FOXP4 in PCa cells

**Fig. 8:**
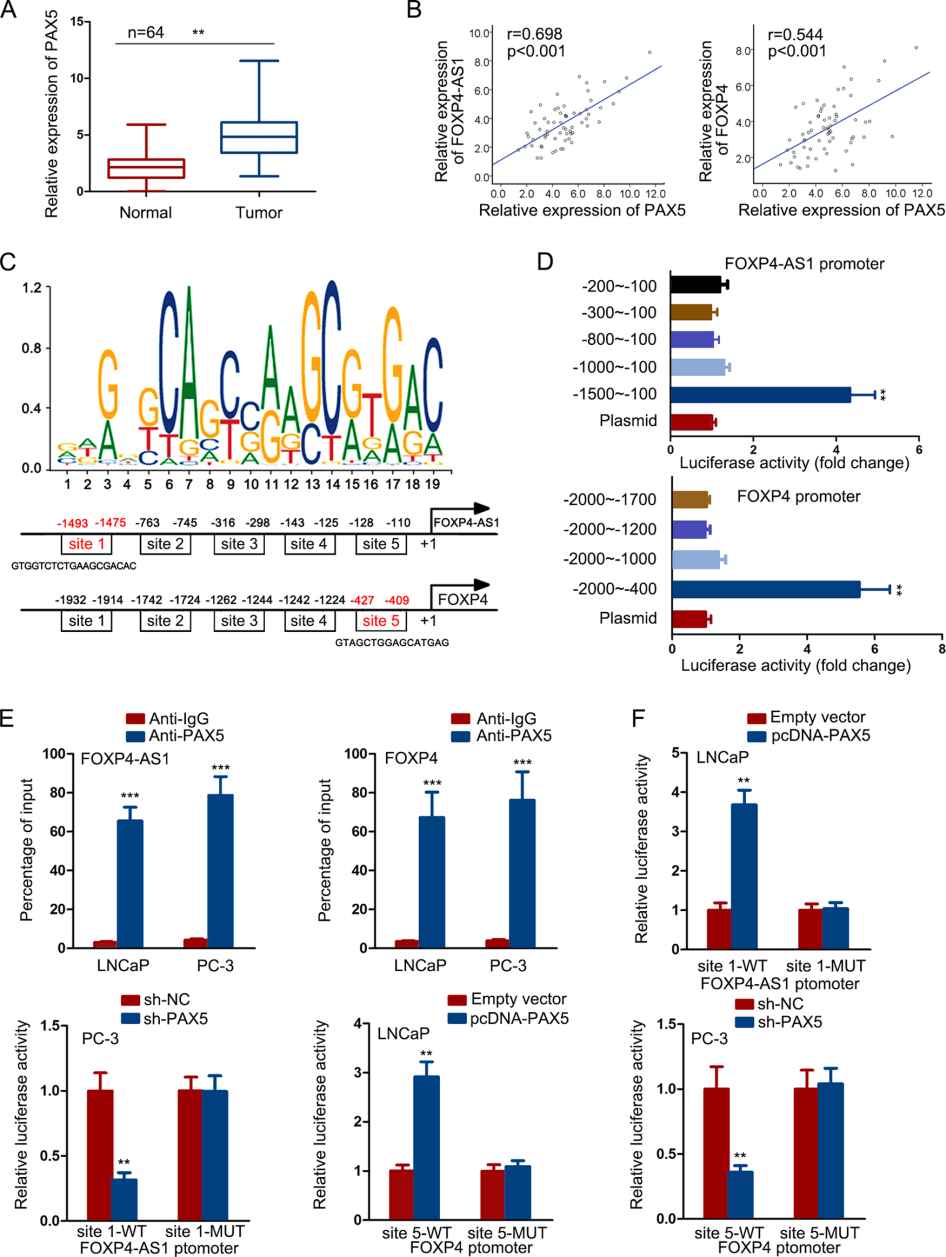
PAX5 transcriptionally activated FOXP4-AS1 and FOXP4 in PCa cells. **a** PAX5 expression in paired PCa samples and normal samples. **b** Positive expression correlation between PAX5 and FOXP4-AS1 or FOXP4 in PCa tissues. **c** DNA motif of PAX5 and predicted five putative binding sites of PAX5 in FOXP4-AS1 or FOXP4 promoter. **d** The luciferase reporter assay demonstrated that site 1 of FOXP4-AS1 promoter (from −1475 to −1493) and site 5 of FOXP4 promoter (from −409 to −427) were potentially responsible for the binding of PAX5 to FOXP4-AS1 or FOXP4 promoter. **e** The ChIP assay showed that there was a strong affinity of PAX5 to FOXP4-AS1 or FOXP4 promoter. **f** Luciferase activity analysis revealed that the luciferase activity of reporter vector containing FOXP4-AS1 promoter or FOXP4 promoter was not changed by PAX5 after mutations in site 1 or site 5. *P < 0.05, **P < 0.01

Transcription activation is an important reason for the dysregulation of genes. In this regard, we detect whether upregulation of FOXP4-AS1 and FOXP4 was caused in this manner. According to the ChIP-seq results of UCSC, we found five potential transcription factors for both FOXP4-AS1 and FOXP4. To analyze the regulatory potential, we overexpressed and silenced them in two PCa cells (Supplementary Fig. 2A, B). Then, we examined the expression change of FOXP4-AS1 and FOXP4 in above two cell lines. The results suggested that PAX5 could positively regulate the expression of both FOXP4-AS1 and FOXP4 in PCa cells (Supplementary Fig. 2C, D). PAX5 was expressed at a high level in PCa samples (Fig. 8a), which was consistent with both FOXP4-AS1 and FOXP4 (Fig. 8b).

Then, we performed loss- or gain-of-function assays to demonstrate the effect of PAX5 on PCa cell proliferation and apoptosis. As presented in Supplementary Fig. 3A, B, upregulation of PAX5 enhanced cell proliferation, while silencing of PAX5 led to the opposite results. In addition, cell apoptosis was negatively regulated by PAX5 (Supplementary Fig. 3C). These findings revealed the oncogenic property of PAX5 in PCa. Furtherly, we investigated whether there is a regulatory relationship between PAX5 and miR-3184-5p. And we found that PAX5 and miR-3184-5p could not regulate each other (Supplementary Fig. 3D, E). To demonstrate the transcriptional regulation of PAX5 on FOXP4-AS1 and FOXP4, we searched DNA motif of PAX5 and predicted five putative binding sites of PAX5 in FOXP4-AS1 or FOXP4 promoter (Fig. 8c). Through the luciferase reporter assay, we found that site 1 of FOXP4-AS1 promoter (from −1475 to −1493) and site 5 of FOXP4 promoter (from −409 to −427) were potentially responsible for the binding of PAX5 to FOXP4-AS1 or FOXP4 promoter (Fig. 8d). To further validate the above results, we separately cloned these putative binding sites into pGL3 vector. As illustrated in Supplementary Fig. 3F. PAX5 overexpression or knockdown affected the luciferase activity of site 1 of FOXP4-AS1 promoter and site 5 of FOXP4 promoter. The ChIP assay showed that there was a strong affinity of PAX5 to FOXP4-AS1 or FOXP4 promoter (Fig. 8e). In order to validate that site 1 of FOXP4-AS1 promoter and site 5 of FOXP4 promoter were actually responsible for the binding of PAX5 to their promoters, we separately mutated these binding sites. Further luciferase activity analysis revealed that the luciferase activity of reporter vector containing FOXP4-AS1 promoter or FOXP4 promoter was not changed by PAX5 after mutations in site 1 or site 5 (Fig. 8f). Collectively, PAX5 acted as a transcription activator for both FOXP4-AS1 and FOXP4 in PCa.

## Discussion

Recently, increasing number of researches has revealed the crucial role of lncRNAs in tumorigenesis^20–22^. Dysregulation of lncRNAs is closely correlated with the overall survival of patients with cancer. For instance, lncRNA MNX1-AS1 and LINC00346 are upregulated in gastric cancer and predicted poor outcome;^23,24^ upregulation of lncRNA EGFR-AS1 indicates low overall survival of patients with renal cancer^25^. LncRNA FOXP4-AS1 has been demonstrated to be a prognostic indicator in osteosarcoma. In this present study, we identified that FOXP4-AS1 was expressed at a high level TCGA PCa samples and collected PCa samples. Similarly, we analyzed whether high expression of FOXP4-AS1 is correlated with the overall survival of patients with PCa. Interestingly. High expression of FOXP4-AS1 is closely correlated with the prognosis of patients with PCa.

Functionally, upregulated lncRNAs can promote cell proliferation and inhibit apoptosis, thereby facilitating tumorigenesis^26–28^. In our current study, we investigated whether FOXP4-AS1 is a regulator in tumorigenesis of PCa. Both gain- or loss-of-function assays were carried out in two PCa cell lines. As expected, high expression of FOXP4-AS1 promoted cell proliferation and suppressed cell apoptosis, indicating the oncogenic property of FOXP4-AS1 in PCa cells. In vivo animal study further demonstrated that FOXP4-AS1 could promoted PCa growth. These data indicated that FOXP4-AS1 exerted oncogenic function in the tumorigenesis of PCa.

Numerous evidence has shown that lncRNAs can exert function in human cancers by regulating their nearby genes^29–31^. According to bioinformatics analysis, we determined that FOXP4 is the nearby gene of FOXP4-AS1. To analyze the potential relationship between FOXP4-AS1 and FOXP4, we examined the expression of FOXP4 in response to the overexpression or knockdown of FOXP4-AS1. We determined the positive regulation of FOXP4-AS1 on FOXP4. More importantly, FOXP4 was also upregulated in PCa samples and cell lines and predicted poor outcome in patients with PCa. Mechanistically, we determined that FOXP4-AS1 was located in the cytoplasm of PCa cells, indicating post-transcriptional regulatory potential of FOXP4-AS1 in PCa. It is widely acknowledged that lncRNAs can regulate gene expression by sequestering miRNAs^32^. In our present study, we investigated whether FOXP4-AS1 can regulate gene expression in this manner. Combining with all these data, we analyzed whether FOXP4-AS1 positively regulated FOXP4 in PCa by serving as a miRNA sponge. Both bioinformatics analysis and mechanism investigation revealed that FOXP4-AS1 and FOXP4 could interact with miR-3184-5p to form a ceRNA network. Through rescue assays, we determined that FOXP4-AS1/miR-3184-5p/FOXP4 axis is an oncogenic pathway in PCa by promoting cell growth.

Upregulation of FOXP4-AS1 and FOXP4 in PCa may be caused by other mechanism, such as transcriptional regulation. In the current study, we found that PAX5 is a transcription factor of FOXP4-AS1 and FOXP4. Consistent with FOXP4-AS1 and FOXP4, PAX5 was also upregulated in PCa tissues. Further mechanism investigation proved that PAX5 enhanced the transcription activity of FOXP4-AS1 and FOX4 by binding to their promoter regions. Therefore, we confirmed that PAX5-induced upregulation of FOXP4-AS1 and FOXP4 contributed to tumorigenesis of PCa.

**Fig. 9:**
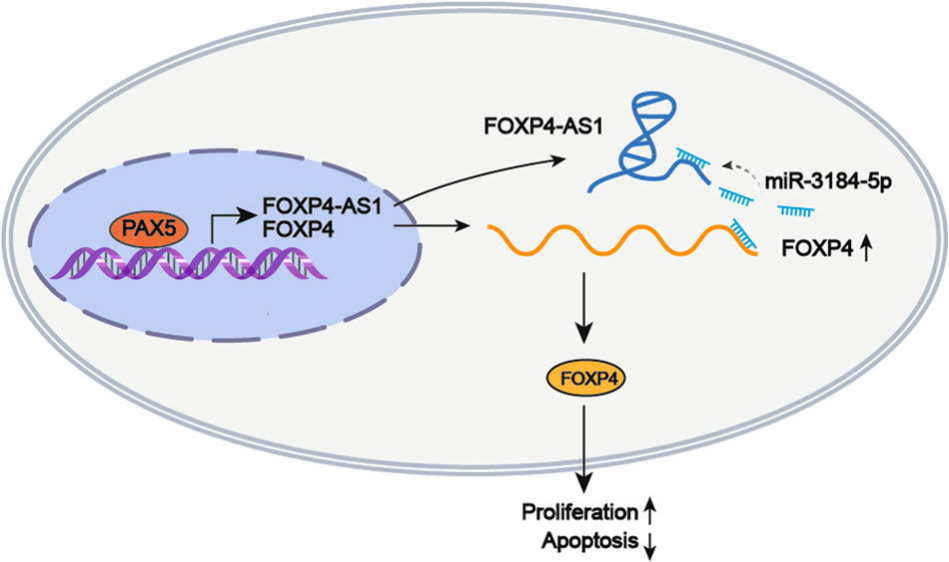
A graphical abstract was created to illustrate the core of this study

In conclusion, our present study demonstrated that FOXP4-AS1 and FOXP4 were upregulated in PCa tissues and cell lines and indicated poor outcome. FOXP4-AS1 upregulated FOXP4 by sequestering miR-3184-5p. MiR-3184-5p was downregulated in PCa samples and predicted poor overall survival. Upregulation of FOXP4-AS1/FOXP4 axis was induced by the transcription activation of PAX5. Thus, this study revealed a novel oncogenic pathway in PCa tumorigenesis (Fig. 9). All our findings may contribute to investigate molecular mechanism associated with PCa tumorigenesis and will provide new thought in exploring the novel diagnostic or therapeutic biomarker for PCa.

## Supplementary information

Supplementary Table 1

Supplementary Figure 1

Supplementary Figure 2

Supplementary Figure 3

Supplementary figure legends
