# Supplementary figures and images for: LncRNA FOXP4-AS1 is activated by PAX5 and promotes the growth of prostate cancer by sequestering miR-3184-5p to upregulate FOXP4
Source: Cell Death Dis. 2019 Jun 17;10(7):472. doi: 10.1038/s41419-019-1699-6 (PMC6572815; doi:10.1038/s41419-019-1699-6)

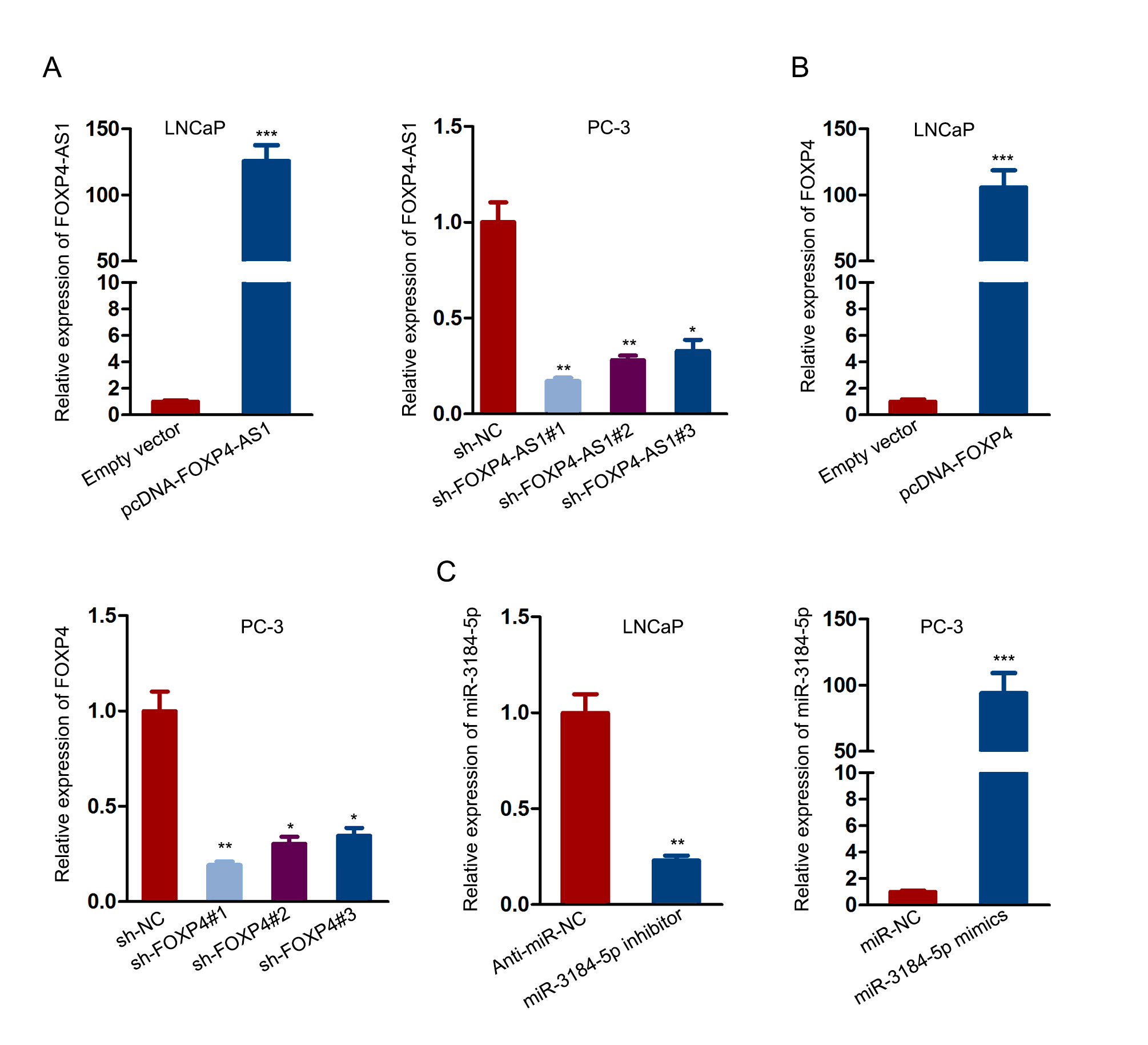

Supplement: Supplementary file 2 — Supplementary Figure 1 [file 41419_2019_1699_MOESM2_ESM.jpg]

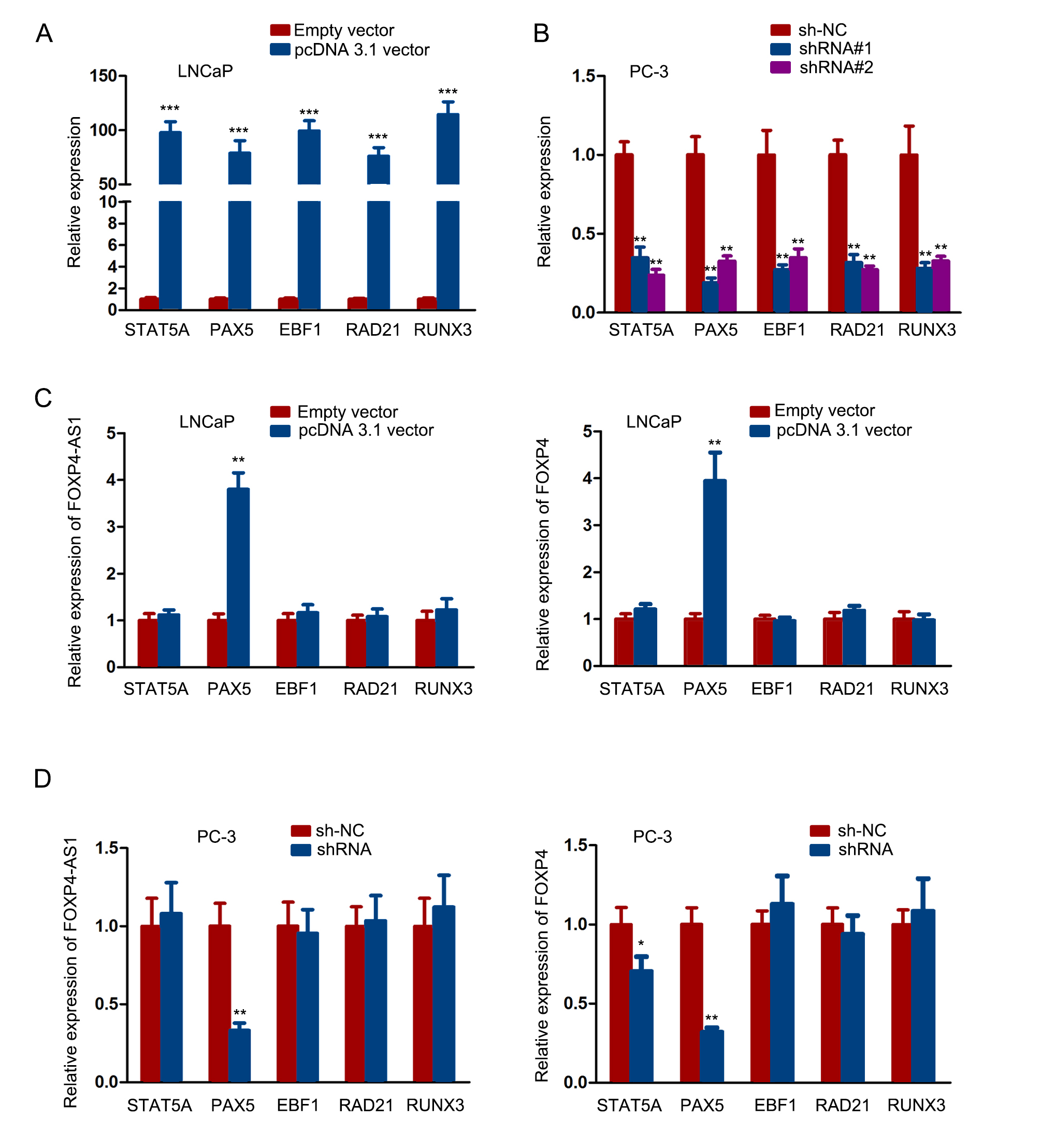

Supplement: Supplementary file 3 — Supplementary Figure 2 [file 41419_2019_1699_MOESM3_ESM.jpg]

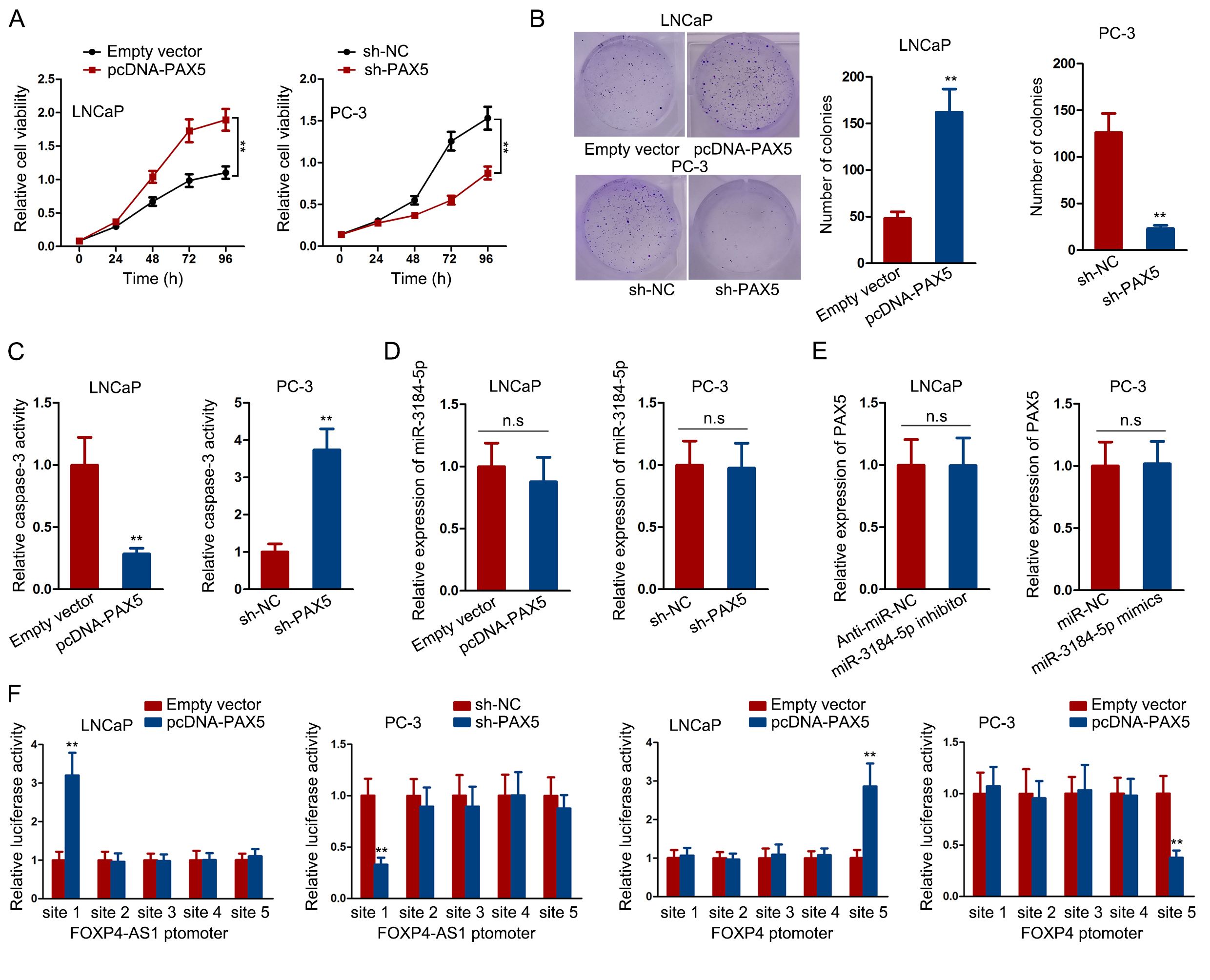

Supplement: Supplementary file 4 — Supplementary Figure 3 [file 41419_2019_1699_MOESM4_ESM.jpg]
