# Supplementary material for: LncRNA FOXP4-AS1 is activated by PAX5 and promotes the growth of prostate cancer by sequestering miR-3184-5p to upregulate FOXP4
Source: Cell Death Dis. 2019 Jun 17;10(7):472. doi: 10.1038/s41419-019-1699-6 (PMC6572815; doi:10.1038/s41419-019-1699-6)
Supplement: Supplementary file 5 — Supplementary figure legends [file 41419_2019_1699_MOESM5_ESM.docx]

**Supplementary figure legends**

**Supplementary Figure 1. A.** Overexpression or knockdown efficiency for FOXP4-AS1 in LNCaP or PC-3 cells. **B.** Overexpression or knockdown efficiency for FOXP4 in LNCaP or PC-3 cells. **C.** LNCaP cell was transfected with miR-3184-5p inhibitor or anti-miR-NC, PC-3 cell was transfected with miR-3184-5p mimics or miR-NC. ^**^P < 0.01, ^***^P < 0.001.

**Supplementary Figure 2. A-B.** Overexpression or knockdown efficiency for five transcription factors in LNCaP cell. **C-D.** The expression level of FOXP4-AS1 and FOXP4 in response to the overexpression or knockdown of five transcription factors. ^*^P<0.05, ^**^P < 0.01, ^***^P < 0.001.

**Supplementary Figure 3. A-B.** Proliferative ability of LNCaP cell transfected with PAX5 expression vector or PC-3 cell transfected with sh-PAX5 was assessed by CCK-8 and colony formation assays. **C.** Caspase-3 activity in PAX5-overexpressed or downregulated cells. **D.** The expression level of miR-3184-5p was tested in LNCaP cell transfected with PAX5 expression vector or PC-3 cell transfected with sh-PAX5. **E.** PAX5 expression in LNCaP cell transfected with miR-3184-5p inhibitor or PC-3 cell transfected with miR-318405p mimics was detected. **F.** The luciferase activity of five putative sites in FOXP4-AS or FOXP4 promoter was evaluated by luciferase reporter assay. ^**^P < 0.01. n.s: no significance.
